# The association between public health engagement in school-based substance use prevention programs and student alcohol, cannabis, e-cigarette and cigarette use

**DOI:** 10.17269/s41997-022-00655-3

**Published:** 2022-07-21

**Authors:** Trish Burnett, Kate Battista, Michelle Butt, Diana Sherifali, Scott T. Leatherdale, Maureen Dobbins

**Affiliations:** 1 School of Nursing, McMaster University, Hamilton, ON Canada; 2 School of Public Health Sciences, University of Waterloo, Waterloo, ON Canada

**Keywords:** Public health, Adolescent, Alcohol drinking, Marijuana smoking, Tobacco smoking, Electronic nicotine delivery systems, Santé publique, adolescent, consommation d’alcool, fumer de la marijuana, fumer du tabac, dispositifs électroniques d’administration de nicotine

## Abstract

**Objective:**

This study examined the associations between public health engagement (PHE) in school-based substance use prevention programs and student substance use. For the purposes of this study, PHE refers to any form of collaboration between the local government public health agency and the school to promote the physical and mental health of students.

**Methods:**

Data for this study were collected from the Cannabis, Obesity, Mental health, Physical activity, Alcohol use, Smoking and Sedentary behaviour (COMPASS) study during the 2018/2019 data collection year. Multilevel logistic regression was used to analyze the associations between PHE and student substance use.

**Results:**

Data from 84 schools and 42,149 students were included; 70% of schools had PHE in substance use prevention programs. PHE in substance use prevention appears to have had no significant impact on student substance use in our models. When PHE was divided into five methods of engagement, it was found that when public health solved problems jointly with schools, the odds of a student using alcohol or cannabis significantly increased. When schools were split into low- and high-use schools for each substance measured, some methods of PHE significantly decreased the odds of cannabis and cigarette use in high-use schools and significantly increased the odds of alcohol and cannabis use in low-use schools.

**Conclusion:**

This study highlights the need to develop better partnerships and collaborations between public health and schools, and the importance of ensuring that school-based substance use prevention programs are evidence-based and tailored to the specific needs of schools and students.

## Résumé

**Objectif:**

L’étude porte sur les associations entre la participation de la santé publique (PSP) aux programmes de prévention de l’usage de substances en milieu scolaire et l’usage de substances par les élèves. Pour les besoins de l’étude, la PSP désigne toute forme de collaboration entre un organisme de santé publique local et une école visant à promouvoir la santé physique et mentale des élèves.

**Méthode:**

Nos données sont tirées de l’étude COMPASS (étude de cohorte sur l’obésité, la consommation de marijuana, l’activité physique, la consommation d’alcool, le tabagisme et le comportement sédentaire) durant l’année de collecte de données 2018-2019. Les associations entre la PSP et l’usage de substances par les élèves ont été analysées au moyen de régressions logistiques multiniveaux.

**Résultats:**

Nous avons inclus les données de 84 écoles et de 42 149 élèves; dans 70 % des écoles, la santé publique participait aux programmes de prévention de l’usage de substances. La PSP à la prévention de l’usage de substances semble n’avoir eu aucun effet significatif sur l’usage de substances par les élèves dans nos modèles. Quand nous avons divisé la PSP en cinq méthodes de participation, nous avons constaté que lorsque la santé publique résolvait les problèmes conjointement avec les écoles, la probabilité qu’une ou un élève consomme de l’alcool ou du cannabis augmentait de façon significative. Quand nous avons divisé les écoles en écoles à faible et à forte consommation pour chaque substance mesurée, certaines méthodes de PSP réduisaient de façon significative les probabilités d’usage de cannabis et de cigarettes dans les écoles à forte consommation et faisaient augmenter de façon significative les probabilités d’usage d’alcool et de cannabis dans les écoles à faible consommation.

**Conclusion:**

Notre étude fait ressortir le besoin de créer de meilleurs partenariats et collaborations entre la santé publique et les écoles, ainsi que l’importance de s’assurer que les programmes de prévention de l’usage de substances en milieu scolaire reposent sur des données probantes et qu’ils sont adaptés aux besoins particuliers des écoles et des élèves.

## Introduction

Substance use in adolescence can have serious lifelong health consequences such as lung disease, cognitive deficits, premature death and future substance use (Camchong et al., 2017; Leslie, 2020; Lubman et al., 2015; World Health Organization, 2018). With the increasing rates of student substance use, it is important to have effective prevention programs in place (Canadian Student Tobacco, Alcohol and Drug Survey, 2019; Hammond et al., 2019). Schools provide an efficient means to reach adolescents while their beliefs and values around substance use are still forming (Faggiano et al., 2014). Many school boards mandate that schools provide substance use prevention programs generally and within the curriculum, but they are not always implemented (Fletcher et al., 2010; Kumar et al., 2013; Ringwalt et al., 2008; Salas-Wright et al., 2019). There is limited research looking into the prevalence of and participation in school-based substance use prevention programs. The research that has been done has found little participation in and availability of evidence-based substance use prevention programs in schools (Fletcher et al., 2010; Kumar et al., 2013; Ringwalt et al., 2008; Salas-Wright et al., 2019).

Collaborations between the health and education sectors have demonstrated positive results in reducing adolescent substance use. For example, research exploring the role of a school health coordinator (O’Brien et al., 2010), a nationwide transdisciplinary health promotion approach (Sigfusdottir et al., 2011) and an online substance use prevention support portal for educators (Stapinski et al., 2017) has demonstrated that health agencies engaging with schools in various ways can help to promote the implementation of substance use prevention programs and support schools in preventing and reducing student substance use. The objective of this study was to determine the impact of public health engagement (PHE) in school-based substance use prevention programs on student substance use. For the purposes of this research, PHE in schools is defined as any form of direct collaboration between the local government public health (PH) agency (e.g., PH unit, local health authority) and the school. A school identified as having PHE in substance use prevention programming would indicate that the school’s local PH agency was involved in developing, implementing, planning, supporting and/or providing resources for programs and/or curricula related to alcohol, cannabis, e-cigarette and/or cigarette use prevention at the school level.

In Canada, each province/territory is responsible for organizing and implementing PH programs and initiatives (Government of Canada, 2020). This is accomplished through various provincial organizations including public health organizations (PHOs) (Government of Canada, 2020). In Alberta for example, there is a fully integrated health system which delivers most health services called Alberta Health Services (AHS). Within AHS, there are health promotion coordinators who work with school boards to promote school health (Alberta Health Services, 2019). In British Columbia (BC), there are five regional health authorities that deliver health services including PH services (The Province of British Columbia, 2019). These authorities are funded by and follow the guidelines set by the Ministry of Health. In Ontario, PH is administered through the Ministry of Health and Long-Term Care (MOHLTC). The MOHLTC identifies PH standards for practice that are implemented by 34 local PH departments (Ministry of Health and Long-Term Care, 2018). This study examined the relationship between PHE in school-based substance use prevention programs and student’s substance use in Alberta, BC and Ontario, controlling for school-level and student-level demographic characteristics.

## Methods

The Cannabis, Obesity, Mental health, Physical activity, Alcohol use, Smoking and Sedentary behaviour (COMPASS) study is an ongoing prospective cohort study (2012–2027). The COMPASS study collects hierarchical longitudinal data from a convenience sample of secondary schools and the students in grades 9 to 12 attending those schools. This study used data collected in Alberta, BC and Ontario during wave seven (2018/2019) of the COMPASS study; 2018/2019 data were used as this was the last wave of data prior to the onset of the COVID-19 pandemic. Student-level data were collected using active-information, passive-consent parental permission protocols. All procedures in the COMPASS study received ethics approval from the University of Waterloo Research Ethics Board (ORE 30118), as well as all participating school board review panels. COMPASS is funded by the Canadian Institutes of Health Research (CIHR) (OOP-110788, MOP-114875, PJT-148562, PJT-149092 and PJT-159693) and Health Canada (#1617-HQ-000012). A full description of the COMPASS study is available online (www.compass.uwaterloo.ca) and in print (Leatherdale et al., 2014).

### Participants

In total, 84 schools and 42,149 students were included in this study. There were eight schools from Alberta (*n* = 3161), 15 from British Columbia (*n* = 9807) and 61 from Ontario (*n* = 29,181). The student response rate was 80.2% and fewer than 5% of students had missing data. Any students with missing data were excluded from the analysis.

### Measures

#### School-level data (School Programs and Policies [SPP] questionnaire)

For each of the school policies and practices measured in the SPP questionnaire, a school administrator who knew the most about the school’s policies and programs was asked about how PH engaged with the school. School administrators were asked “During the past 12 months, what role did your local Public Health Unit play when working with your school on addressing [specific health behaviour] for students?” School administrators were asked to select all methods that applied from the following four options: (1) ‘No contact with local Public Health Unit’; (2) ‘Provided information/resources/programs (e.g., posters, toolkits)’; (3) ‘Solved problems jointly’; and (4) ‘Developed/implemented program activities jointly’. If school administrators selected the first method, the school was defined as having no engagement with PH. If method two, three and/or four were selected, the school was defined as having any engagement PH. School administrators were also asked: “Other than classes/curriculum, does your school offer any programs that address [specific health behaviour] prevention?” If school administrators answered ‘yes’, then the follow-up question “Who runs these programs?” was asked. School administrators could select from three options: (1) ‘Programs run by school’ (2) ‘Programs run by external organization’; and (3) ‘Programs run by Public Health Unit’. If school administrators selected option three, the school was analyzed as having any engagement with PH. Furthermore, the impact of each method of PHE on student substance use was also assessed (see Table 1).

**Table 1:** Method of public health engagement definitions

Method of public health engagement	Description of public health unit engagement with schools reported by school administrators
Method 1	‘Provided information/resources/programs (e.g., posters, toolkits)’
Method 2	‘Solved problems jointly’
Method 3	‘Developed/implemented program activities jointly’
Method 4	‘Programs run by Public Health Unit’

#### School-level data (Statistics Canada data)

Neighbourhood-level characteristics surrounding e2ach participating school were collected from the 2016 Canadian Census using postal codes within corresponding school boundaries. This information provides data on the urbanicity and socioeconomic status (SES) of the school (Zuckermann et al., 2019). Schools in a location with a population of 100,000 or greater and a population density of at least 400 per square kilometre were defined as large urban. Schools in a location with a population between 30,000 and 99,999 and a population density of at least 400 per square kilometre were defined as medium urban, and schools in a location with a population less than 30,000 or a population density under 400 per square kilometre were defined as small urban/rural. The median household income for the school catchment area acted as a proxy for SES (Statistics Canada, 2020). School enrollment data for the 2018/2019 academic year for each participating school were also collected (see Table 2).

**Table 2:** Descriptive statistics

		Schools		Students	
		*n*	%	*n*	%
Categorical school-level variables					
Province	Alberta	8	9%	3161	8%
	British Columbia	15	18%	9807	23%
	Ontario	61	73%	29,181	69%
Urbanicity	Large urban	37	44%	24,110	57%
	Medium urban	14	17%	7235	17%
	Small urban/rural	33	39%	10,804	26%
Public health engagement in alcohol and cannabis use prevention	No engagement	30	36%	14,223	34%
	Engagement	54	64%	27,926	66%
Public health engagement in e-cigarette and cigarette use prevention	No engagement	28	33%	12,928	31%
	Engagement	56	67%	29,221	69%
Total		84	100%	42,149	100%
Continuous school-level variables		*M*	*SD*	*M*	*SD*
School median income (’000s)		72.66	16.17	72.95	17.64
School enrolment size (’00s)		5.28	2.57	6.51	2.65
Student-level variables				*n*	%
Grade	9			11,029	26%
	10			11,434	27%
	11			10,668	25%
	12			9018	21%
Sex	Female			20,893	50%
	Male			21,256	50%
Ethnicity	White			24,750	59%
	Black			1822	4%
	Asian			6770	16%
	Hispanic			1355	3%
	Other/mixed			7452	18%
Substance use	Current alcohol user			12,103	29%
	Current binge drinker			7110	17%
	Current cannabis user			6768	16%
	Current e-cigarette user			12,135	29%
	Current cigarette user			3265	8%
Total				42,149	100%

#### Student-level data (COMPASS questionnaire)

Five questions were used to measure student alcohol, cannabis, e-cigarette and cigarette use. To assess current drinking, students were asked “In the last 12 months, how often did you have a drink of alcohol that was more than just a sip?” Students were asked to respond with one of ten options from ‘I have never drunk alcohol’ to ‘every day’. A student was considered a current drinker if they reported having a drink of alcohol at least once a month that was more than just a sip. To assess binge drinking, students were asked “In the last 12 months, how often did you have 5 drinks of alcohol or more on one occasion?” Students were asked to respond with options from ‘I have never done this’ to ‘daily or almost daily’. A student was considered a current binge drinker if they reported having five or more drinks on one occasion at least once a month. To measure cannabis use, students were asked “In the last 12 months, how often did you use marijuana or cannabis (a joint, pot, weed, hash)?” Students were asked to respond with one of the nine options from ‘I have never used marijuana’ to ‘every day’. A student was considered a current cannabis user if they reported using cannabis at least once a month. To measure e-cigarette and cigarette use, students were asked “On how many of the last 30 days did you use an e-cigarette?” and “On how many of the last 30 days did you smoke one or more cigarettes?” Students were asked to respond with one of eight options from ‘None’ to ‘30 days (every day)’. A student was considered a current e-cigarette or cigarette user if they reported smoking an e-cigarette or cigarette at least once in the last 30 days, respectively.

This study also considered self-reported demographic data collected by the COMPASS student questionnaire. At the beginning of the student questionnaire, students were asked questions to describe themselves. This study used data collected from the following questions: “What grade are you in?”, “Are you male or female?”, “How would you describe yourself? (1) ‘White’, (2) ‘Black’, (3) ‘Asian’, (4) ‘Hispanic’, (5) ‘Other/Mixed’” (see Table 2).

## Data analysis

Descriptive statistics were conducted initially; counts, percentages and chi-square tests were calculated for dichotomous and categorical variables. Mean, standard deviation and *t*-tests were calculated for the continuous variables. The percentage of schools reporting PHE in each of the health domains measured (physical activity, healthy eating, bullying, sedentary behaviour, mental health, tobacco and e-cigarette use, and alcohol and cannabis use) was then calculated. To determine what school characteristics were associated with a school reporting PHE in (a) alcohol and/or cannabis use and (b) e-cigarette and/or cigarette use prevention programming at their school, chi-square tests were done with the categorical school-level variables (province and urbanicity) and *t*-tests with the continuous variables (SES and school size). Each school-level variable was tested for associations with PHE in alcohol and/or cannabis use prevention programming and e-cigarette and/or cigarette use prevention programming. SAS version 9.4 was used to analyze the data and the alpha was set to 0.05.

The first part of this analysis included all participating schools together and in the second part, schools were separated into low- or high-use schools based on student substance use. The percentage of students using alcohol, cannabis, e-cigarettes and cigarettes was calculated for each school. Students attending schools which reported substance use rates below the mean rate of substance use were analyzed in the low-use group and students from schools above the mean were analyzed in the high-use group. To determine whether PHE is associated with the likelihood of student substance use, controlling for school-level and student-level demographic characteristics, student- and school-level data were analyzed with a multilevel logistic regression analysis. Generalized estimating equations (GEE) with exchangeable working correlation were used to cluster by school. This analysis using GEE accounts for the clustering of similar students within a school (Fitzmaurice & Ravichandran, 2008), based on the assumption that students within the same school may be more alike compared to students from other schools. A GEE is used to estimate the parameters of a regression model when a potential correlation exists between subjects. GEE methods are robust even when the covariance structure is mis-specified and can therefore account for dependence between subjects, even when the exact structure of the correlation is unknown (Fitzmaurice & Ravichandran, 2008).

## Results

Student characteristics of the 2018/2019 sample are presented in Table 2.

### Any health domain

Overall, 87% of schools reported some form of PHE in at least one health domain. Only 34% of schools reported PHE in all 7 health domains (see Table 3).

**Table 3:** Frequency of schools indicating public health engagement in the health domains

	Schools	
	*n*	%
Schools indicating public health engagement in any domain		
No	11	13%
Yes	73	87%
Total	84	100%
Number of domains within a school with reported public health engagement		
1	9	12%
2	3	4%
3	8	11%
4	5	7%
5	12	16%
6	11	15%
7	25	34%
Total	73	100%
Frequency of schools indicating public health engagement in each domain		
Physical activity	57	68%
Healthy eating	60	71%
Bullying	40	48%
Sedentary behaviour	34	40%
Mental health	58	69%
Tobacco and e-cigarettes	56	67%
Alcohol and marijuana	54	64%

### Alcohol and/or cannabis use prevention programming

We identified that 64% of schools (*n* = 54) reported PHE in alcohol and/or cannabis use prevention programming (see Table 2). Among the schools reporting engagement, most schools (44%, *n* = 24) reported that PH provided information/resources/programs (see Table 4).

**Table 4:** The method of PHE in substance use prevention programming reported at schools with PHE

	Alcohol and/or cannabis use prevention programming reported at schools		E-cigarette and/or cigarette use prevention programming reported at schools	
	*n*	%	*n*	%
Method of engagement:				
Provided information/resources	24	44%	23	41%
Solved problems jointly	8	15%	8	14%
Developed/implemented programs jointly	7	13%	10	18%
Programs run by public health unit	15	28%	15	27%
Total	54	100%	56	100%

There were no statistically significant differences in alcohol and cannabis use overall when no engagement with PH was compared to any method of engagement across all schools (see Table 5). However, a student attending a school where PH solved problems jointly had higher odds of binge drinking (*AOR* = 1.51, 95% CI [1.24, 1.83], *p* < 0.001), alcohol use (*AOR* = 1.46, 95% CI [1.22, 1.74], *p* < 0.001) and cannabis use (*AOR* = 1.37, 95% CI [1.07, 1.74], *p* = 0.012) compared to a similar student from a school with no PHE (see Table 5).

**Table 5:** Public health engagement in substance use prevention and programming and student substance use

Method of public health unit engagement	All schools			Low-use schools			High-use schools		
	*AOR*	95% CI	*p*-value	*AOR*	95% CI	*p*-value	*AOR*	95% CI	*p*-value
Current binge drinking									
No engagement (ref)									
Any engagement	1.07	0.88, 1.31	.487	0.85	0.69, 1.05	.14	1.04	0.86, 1.26	.692
Method 1	0.98	0.78, 1.23	.829	0.78	0.61, 1.00	.047*	1.02	0.84, 1.23	.871
Method 2	1.51	1.24, 1.83	<.001*	1.61	1.26, 2.05	<.001*	1.11	0.87, 1.41	.408
Method 3	1.08	0.74, 1.58	.69	0.87	0.53, 1.42	.571	1.07	0.79, 1.45	.639
Method 4	0.93	0.66, 1.29	.648	0.92	0.73, 1.16	.482	0.97	0.69, 1.38	.876
Current alcohol drinking									
No engagement (ref)									
Any engagement	1.09	0.91, 1.30	.331	0.96	0.80, 1.17	.701	1.00	0.86, 1.17	.952
Method 1	0.98	0.79, 1.21	.866	0.85	0.69, 1.04	.121	1.00	0.85, 1.18	.992
Method 2	1.46	1.22, 1.74	<.001*	1.59	1.30, 1.94	<.001*	1.07	0.88, 1.29	.515
Method 3	1.09	0.80, 1.49	.577	1.05	0.72, 1.55	.793	0.98	0.73, 1.31	.877
Method 4	1.00	0.77, 1.29	.988	1.08	0.88, 1.33	.45	0.95	0.74, 1.23	.698
Current cannabis use									
No engagement (ref)									
Any engagement	1.02	0.88, 1.18	.79	1.04	0.83, 1.31	.713	0.99	0.84, 1.17	.907
Method 1	0.88	0.74, 1.04	.13	0.89	0.71, 1.12	.315	0.97	0.84, 1.11	.622
Method 2	1.37	1.07, 1.74	.012*	1.12	0.81, 1.54	.502	1.07	0.79, 1.45	.647
Method 3	0.96	0.80, 1.16	.689	1.26	1.00, 1.59	.049*	0.82	0.69, 0.98	.026*
Method 4	1.04	0.83, 1.30	.755	1.23	0.90, 1.67	.202	0.98	0.85, 1.13	.812
Current e-cigarette use									
No engagement (ref)									
Any engagement	1.00	0.86, 1.16	.983	0.93	0.77, 1.13	.474	1.04	0.90, 1.20	.564
Method 1	0.96	0.81, 1.14	.62	0.90	0.72, 1.11	.31	1.02	0.85, 1.22	.844
Method 2	1.08	0.85, 1.36	.529	0.79	0.61, 1.01	.062	1.05	0.90, 1.23	.545
Method 3	0.96	0.78, 1.18	.689	1.01	0.78, 1.30	.957	0.99	0.79, 1.25	.958
Method 4	1.04	0.83, 1.30	.72	0.99	0.78, 1.25	.936	1.09	0.90, 1.31	.375
Current cigarette use									
No engagement (ref)									
Any engagement	0.90	0.68, 1.18	.431	0.92	0.72, 1.17	.505	0.79	0.64, 0.99	.04*
Method 1	0.82	0.61, 1.10	.182	0.92	0.70, 1.20	.527	0.74	0.58, 0.93	.011*
Method 2	1.13	0.75, 1.71	.554	0.95	0.74, 1.23	.71	0.92	0.67, 1.27	.615
Method 3	0.89	0.63, 1.27	.53	0.64	0.41, 1.01	.054	0.77	0.57, 1.03	.083
Method 4	0.84	0.57, 1.23	.362	1.03	0.75, 1.41	.846	0.75	0.52, 1.07	.11

Note. *AOR* adjusted odds ratio, *CI* confidence interval, *Method 1* provided information/resources, *Method 2* solved problems jointly, *Method 3* developed/implemented programs jointly, *Method 4* programs run by public health unit

When schools were divided into high- and low-use schools, this study found a student attending a low-use school where PH provided information/resources/programs had lower odds of binge drinking compared to a similar student from a low-use school with no PHE (*AOR* = 0.78, 95% CI [0.61, 1.00], *p* = 0.047). A student from a low-use school where PH solved problems jointly had greater odds of binge drinking compared to a similar student from a low-use school with no PHE (*AOR* = 1.61, 95% CI [1.26, 2.05], *p* < 0.001). A student attending a low-use school where PH solved problems jointly had greater odds of alcohol use compared to a similar student from a low-use school with no PHE (*AOR* = 1.59, 95% CI [1.30, 1.94], *p* < 0.001). A student attending a low-use school where PH developed/implemented program activities jointly had greater odds of using cannabis compared to a similar student from a low-use school with no PHE (*AOR* = 1.26, 95% CI [1.00, 1.59], *p* = 0.049). Whereas in high-use schools, a student attending a school where PH developed/implemented program activities jointly had lower odds of using cannabis compared to a similar student from a high-use school with no PHE (*AOR* = 0.82, 95% CI [0.69, 0.98], *p* = 0.026) (see Table 5).

### E-cigarette and/or cigarette use prevention programming

Overall, 67% of schools (*n* = 56) reported PHE in e-cigarette and/or cigarette use prevention programming (see Table 2). Of the schools reporting engagement, most schools (41%, *n* = 23) reported that PH provided information/resources/programs (see Table 4).

There were no statistically significant associations observed for any method of PHE and e-cigarette and cigarette use. However, when schools were divided into low- and high-use schools, a student attending a high-use school where PH provided information/resources/programs had lower odds of using cigarettes compared to a similar student from a high-use school with no PHE (*AOR* = 0.74, 95% CI [0.58, 0.93], *p* = 0.011). Second, a student attending a high-use school reporting any method of PHE had lower odds of using cigarettes compared to a similar student from a high-use school with no PHE (*AOR* = 0.79, 95% CI [0.64, 0.99], *p* = 0.04). There were no significant associations between cigarette use and other methods of PHE (see Table 5). It is likely the significant finding in any engagement was driven by the significant association seen when PH provided information/resources/programs.

## Discussion

The goal of this study was to measure PHE in schools and the impact on student substance use. We found that more than 1 in 10 schools reported no PHE in any health domain, and even among the schools reporting PHE, two thirds did not have PHE across all health domains. These results draw attention to the fact that there are schools with no PHE and in the schools with some form of engagement, there is variability in the number of health domains in which PH was engaged (see Table 3). Overall, there appears to be an opportunity to improve PHE with schools with respect to all healthy behaviour programming.

PHE in schools did not have a statistically significant impact on student substance use generally, although in some instances it appeared it may increase use (see Table 5). When schools were divided into low- and high-use schools, in general, PHE in low-use schools increased the odds of student alcohol and cannabis use whereas PHE in high-use schools demonstrated a reduction in the odds of a student using cannabis and smoking cigarettes (see Table 5). These results suggest the rate of substance use within the school is important and may impact the effectiveness of PHE on substance use. This is consistent with other research which has found that certain school-based prevention programs achieve greater results when focused on high-risk populations compared to the general student population (Onrust et al., 2016).

This study also examined the impact of different methods of PHE (see Table 1). When PH provided information/resources/programs, binge drinking in low-use schools and cigarette use in high-use schools was reduced and when PH developed/implemented program activities jointly, cannabis use in high-use schools was reduced (see Table 5). When programs were run by PH, there were no statistically significant results associated with a change in any substance use. This suggests that even if the PH agency is not running the program there is still a benefit when schools work with PH agencies to obtain information/resources or jointly plan and implement programs. Research on the effectiveness of school-based substance use has found that social competence and combined approaches (including aspects of social competence, social norms and knowledge-based approaches) have demonstrated the greatest effect in reducing student substance use (Faggiano et al., 2014; MacArthur et al., 2016; Onrust et al., 2016; Thomas et al., 2013; Thomas et al., 2015). However, the quality and quantity of the information and guidance that was provided to the school regarding substance use prevention in this study is unknown. It is also unknown whether other government agencies/departments or other advocacy groups (separate from PH) are working with the schools on substance use prevention. These groups may not have been captured in this study; however, PHOs should be aware of what other programs are being carried out in schools. It is important that both PHOs and any other health agency working with schools encourage the use of evidence-based substance use prevention programming and consider both the method of engagement and the risk level of the students to achieve the greatest effect on student substance use.

Future research may consider examining the relational aspect of PHE with schools to determine its impact over time on the effectiveness of PHE on student substance use. Future research could also examine PHE at the school board level. This would achieve a greater understanding of the impact of PHE in schools and further guide PHOs on where to focus their limited resources. Future research may also consider examining prevention programs for each substance separately to provide greater clarity as to the effectiveness of the prevention program and the impact of PHE in schools. Finally, future research and surveillance on the types of prevention programs being administrated and whether these programs are evidence-based would also provide a valuable indication of the quality of substance use prevention programs in schools.

Results from this study should be interpreted in the light of some limitations. First, it was not possible to determine the type, duration and intensity of the prevention programs carried out at the schools or the quality of the information and guidance provided by PH from the data collected. This prevents an examination of which programs produce which effects (no effect, reduced use, increased use). Second, this study was limited to measuring direct engagement between the local government PH agencies and the school. These results would not capture the impact of PHE at the provincial and school board level or prevention programs run by other health agencies separate from PH. It is also possible that PHE took place in broader health promotion programs at the school that may have been missed by measuring PHE in programs targeting a specific behaviour. Third, the question used to measure PHE on the SPP combined alcohol and cannabis use prevention programs together and e-cigarette and cigarette use prevention programs together. Therefore, it is unknown whether the school prevention program was focused on one of these substances or both. Fourth, the student-level data were self-reported and are therefore subject to a risk of under-reporting, social desirability, and recall bias (Biemer & Witt, 1997). However, the student questionnaire used is based on previously validated measures to help mitigate the risk of bias (Wong et al., 2012). Finally, this study was a cross-sectional survey and therefore could not establish temporality or causality between PHE and student substance use.

## Conclusion

The results of this study showed there is an opportunity for greater public health engagement in all health programs at schools and highlighted the importance of the method of engagement and the risk level of the school population in substance use prevention programs. The knowledge gained from this study can be used to inform guidelines set out by PHOs to improve collaboration between the health and education sectors and facilitate the implementation of effective school-based substance use prevention programs to better prevent and reduce student substance use.

## Contributions to knowledge

What does this study add to existing knowledge?

- There are many local schools and health domains that do not receive any public health engagement.
- Overall public health engagement in school-based substance use prevention programming was not statistically significantly associated with student substance use.
- The impact of public health engagement in school-based substance use prevention programs on student substance use varied across schools based on the method of engagement and the rate of substance use at the school.

What are the key implications for public health interventions, practice or policy?

- There is an opportunity for greater public health engagement in more schools across more health domains.
- Public health agencies should consider the rate of substance use at the school when engaging with schools in substance use prevention—there is evidence to suggest focusing public health engagement in schools with higher rates of substance use.
- Public health engagement where programs were run by public health did not result in lower levels of student substance use; therefore, it may be valuable for public health to focus on providing information/resources/programs, solving problems jointly or developing/implementing program activities jointly with schools.
